# Elite Adolescent Ice Hockey Players: Analyzing Associations between Anthropometry, Fitness, and On-Ice Performance

**DOI:** 10.3390/ijerph19158952

**Published:** 2022-07-23

**Authors:** Gaëtan Martini, Jean-François Brunelle, Vincent Lalande, Jean Lemoyne

**Affiliations:** 1 Ice Hockey Research Laboratory, Université du Québec à Trois-Rivières, Trois-Rivières, QC G8Z 4M3, Canada; jean-francois.brunelle@uqtr.ca (J.-F.B.); vincent.lalande@uqtr.ca (V.L.); jean.lemoyne@uqtr.ca (J.L.); 2 Department of Human Kinetics, Université du Québec à Trois-Rivières, Trois-Rivières, QC G8Z 4M3, Canada; 3 Physical Activity and Sports Center, Université du Québec à Trois-Rivières, Trois-Rivières, QC G8Z 4M3, Canada

**Keywords:** body mass, height, fitness assessment, on-ice testing, young athletes, selection camps, match performance, talent identification

## Abstract

In the field of ice hockey, body mass and height have always played a role in team selection. This study aims to analyze the associations between anthropometry and multiple dimensions of performance among elite adolescent ice hockey players. Methods: 187 adolescent players (males: *n* = 80, 13.81 years; females: *n* = 107, 14.96 years) took part in the study, in Camp 1. Off-ice fitness and on-ice skating tests were performed. Camp 2 consists of on-ice tests and match performance with players selected by coaching staff at Camp 1. Camp 2 data came from official matches performance and a combination of skating tests and intrasquad matches. Hockey Quebec’s selection process consists of going from an entire pool of athletes to a team of twenty-five players, through several camps spread over two years. Correlation analyses were conducted to verify associations between anthropometric measures and performances. Results: In Camp 1, heavier-taller male athletes displayed better performances in most off-ice fitness tests, while heavier female athletes tended to display poorer performance. Camp 2 showed no significant correlations in on-ice tests and match performance. However, some tendencies were observed: heavier male players were less involved in blocked shots, and taller females were more agile. Conclusions: Despite off-ice advantages, the taller-heavier prototype does not translate directly to better performance on the ice among elite adolescent players. Coaches should analyze anthropometric characteristics with caution, and place more focus on match-specific abilities to identify talent.

## 1. Introduction

Ice hockey is a high-intensity team sport that requires sport-specific technical skills [1], as well as physical attributes such as strength, speed, and agility [2]. Ice hockey has evolved significantly at the international scene. In fact, there are now more than 1.5 million players worldwide, in more than 75 countries [3]. Consequently, ice hockey has raised scholars’ interests and has consequently led to an increase in yearly scientific publications [4]. Since the last decade, the internationalization of hockey has led to an intensification of the physiological, muscular, and technical-tactical requirements to perform, both for professionals [5,6] and youth [7,8]. To reach the elite level, young athletes (e.g., 14–18 years old) need to develop their technical, physical, and tactical qualities, by optimizing on-ice training and physical preparation [9,10]. The late adolescence stage, which generally occurs after the pubertal growth peak, is a crucial stage in the development of advanced expertise [11]. Through the developmental model for sports such as ice hockey, organizations and coaches are interested in knowing more about the determinants of performance contributing to success, to propose adapted content [12]. With this long-term developmental approach in mind, talent identification processes have been developed [13,14], based on the assessment of anthropometric characteristics (e.g., body mass, height, body mass index); body composition (i.e., body fat percentage, lean mass rate) [15,16]; as well as performance, specifically, power–strength–velocity performances in jumping, sprinting and/or skating, and throwing/throwing [17,18]. Therefore, the stakeholders involved in team selection (and player evaluation) processes rely on all or on part of these precise, comprehensive, and complex evaluation methods [19,20], while considering the modern evolution of the sport and the associated standards [5]. Past research suggests that anthropometric characteristics and/or body composition are variables associated with success in sport, whether at the senior professional level [21,22], or among younger competitive athletes [23,24]. In team sports such as basketball, handball, or rugby, characteristics such as body mass, height, and wingspan are among the factors that determine an athlete’s potential [25,26,27]. There is a plethora of methods for measuring the anthropometric characteristics of athletes. Three of them are regularly used in ice hockey research: the “traditional” method with a scale and a stadiometer [28,29]; skinfold method, to estimate free fat mass [30,31]; and dual energy X-ray absorptiometry, which is referenced as the gold standard in anthropometric assessment methods [32,33]. Of all the existing techniques regularly used in ice hockey, the traditional measurement method is the least complex and the quickest to implement with a population of adolescents, especially considering the rapid morphological changes that take place during this developmental stage.

Considering the importance of physical dominance in ice hockey, evaluators have always placed considerable importance on this facet of talent appraisal [34]. The most well-known evaluation tool in ice hockey is the National Hockey League (NHL) Combine, where high-potential young athletes, also known as “prospects”, are tested on various performance components, including anthropometric characteristics [35]. Quinney et al. [36] provide longitudinal insight into the changing anthropometric characteristics of professional ice hockey players. It is reported that height and body mass have followed similar trends over the years, with a slight increase between 1980 and 2005 [37]. The anthropometric profile of high-level ice hockey players also varies according to player position, with defensemen reported to be taller and heavier than forwards, findings corroborated by Chiarlitti et al. [16]. Body composition has been further associated with performance, as it has been shown that the most successful teams (i.e., successful teams in international competitions) are statistically the most athletic (higher body mass in relation to higher lean muscle mass) [38]. However, the association between anthropometric profile and performance in developing hockey players has its challenges. Leiter and Cordingley are among the few (if not the only) that have looked at the analysis of anthropometric characteristics in adolescent ice hockey players [9,39]. Their results showed that most morphological changes (mainly height and body mass) occur between thirteen and fifteen years, compared to older athletes of sixteen to seventeen years old. Despite the importance given to anthropometry in competitive sport, the literature has focused little on the relationship between these characteristics, physical qualities, on-ice performance, and how it could influence the selection of young athletes in ice hockey [12,34]. 

In ice hockey, performance is measured according to several components: functional off-ice testing or “off-ice fitness” [40]; on-ice physical and technical skills or “on-ice skating abilities” [17]; repeated intense sequences situations on the ice [41]; in-situ match time conditions [42] or in longitudinal follow-up during the season [43]. It has been established that the NHL Combine [44], mentioned above, gathers the most talented professional athletes to assess them on various components. Players are subjected to various evaluations including: a complete medical and orthopedic examination (history, electrocardiogram, and echocardiogram); a battery of physical tests assessing neuromuscular qualities (strength, power, muscular endurance, and agility); physiological qualities (anaerobic and aerobic power); as well as anthropometric characteristics and body composition [35].

For young players, national team talent evaluation camps are opportunities to measure performance indicators in various contexts. These events, organized by the federations, serve, among other things, to periodically evaluate athletes with the purpose of getting more information about their development. Selection camps help federations select athletes who will represent the country in the national youth teams (under 18 and under 20) [12]. These unique moments also contribute to the long-term development of young athletes, a complex process in ice hockey to reach the elite level [11], by offering them standardized evaluation procedures and personalized quality coaching. To prepare for this, and to meet professional hockey requirements, young athletes are encouraged to improve their physical condition and their on-ice skills [10]. As it was shown by Nightingale et al. [45], athletes’ fitness assessment is a topic that has been extensively examined over the last twenty years. The systematic review by Huard-Pelletier and colleagues provides us with an overview of the relationships between the different aspects of performance mentioned above. The relationships between physical fitness tests and on-ice performance (skating and match) are relatively well established and accepted [13], whether at the professional or youth level. In team sports, interactions exist between anthropometry, performance, and selection of adolescent athletes [46,47]. Ice hockey is a late maturity sport, where talent identification is a complex process [48]. Evaluation of anthropometric characteristics plays a role in the selection process [35,38,49], as does the performance on the ice [7,29] in elite players. However, assessment of these variables, their relationship to performance and talent identification in young hockey players, has been less studied [12,34]. On one hand, the physical condition of young hockey players aged from thirteen to sixteen years improves as anthropometric variables (height and body mass) increase, compared to older athletes where only physical fitness varies [9,39]. On the other hand, some authors have shown that height or body mass may influence the selection process during detection camps for youth hockey players [12]. Results indicate that anthropometric characteristics, such as body mass, could negatively affect ice hockey performance in female athletes [50], a hypothesis recently confirmed by Lemoyne and colleagues [14].

### Objectives of the Study

Regarding the potential associations between anthropometry and performance in ice hockey, a few studies have investigated the links between off-ice functional fitness, on-ice fitness, and their potential to link it with performance [7,12,29,34,50]. Indeed, most of these studies were conducted with small samples, which limits the generalizability of results. To our knowledge, no article has evaluated such associations, most specifically in the team selection process. Moreover, considering each gender separately is relevant because the correlations might differ. This study aims to understand the associations between anthropometric measures and the performance of young ice hockey players during a selection process, from the entire pool of players under 16 years old in a Canadian province to a final team of 25 players who will compete at a national tournament. This study has three specific objectives: (i) to assess the evolution of anthropometry over a crucial selection period (5 months) in preparation for a U16 provincial team selection; (ii) to verify the relationships between anthropometric measures and performance in the context of off-ice physical testing, on-ice performance testing, and match performance; and (iii) to analyze the possible effects of gender and playing position on anthropometric and performance data.

## 2. Materials and Methods

### 2.1. Participants—Procedures

#### 2.1.1. Participants

**Figure 1:**
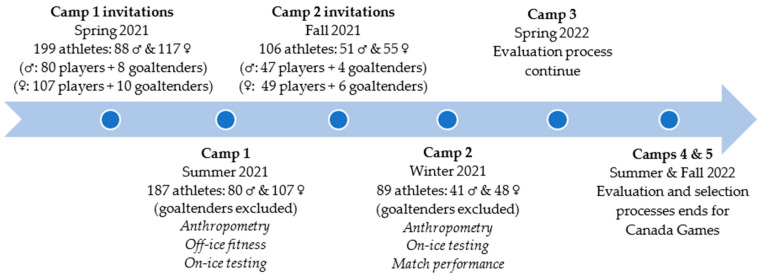
Schedule timeline of Hockey Quebec selection process, and assessment fields during Camps 1 and 2.

This study was developed in collaboration with researchers and the governing bodies of Hockey Quebec, the province’s ice hockey federation. The present investigation refers to a two-phase selection process that occurs through a 5-month period. Figure 1 synthetizes the process conducted in two evaluation camps. In Camp 1, the purpose was to pre-select each cohort’s best 45 players. In the long term, the team selection process terminated in fall 2022 (e.g., two teams of 20 players). In **Camp 1**, a total of 199 players between the ages of 14 and 16 years (86 boys: 43%, 14 years old; 113 girls: 57%, 16 years old) were invited to the Team Quebec evaluation camp. The criteria for invitation were based on players’ regular season performances as well as evaluation from the province’s hockey program directors. Indeed, both prospect (e.g., male and female) camps serve as an important stage to determine those who will represent Quebec in national competitions and potentially be invited to Team Canada evaluation camps in the following years. A week before each camp, players were informed about the research project during an online information meeting. Those who agreed to participate were asked to sign a consent form (if <16 years old, the parents signed). The project was approved by the ethics board of the researchers’ institution (CER-21-278-07.09). The full protocol has been published in a previous article (see Lemoyne et al. [14]). The testing procedure was completed on three separate days. **Camp 2** was performed five months later, with two different contexts (setting and structural organization of the camps), according to gender. For the male cohort, 45 athletes (selected from Camp 1) were invited to take part in a provincial tournament-showcase. For the female cohort, no competitions were organized. The provincial ice hockey federation decided to organize a second evaluation camp (on-ice testing, training sessions, intra-squad matches) in which 48 female athletes took part. Camp 2 was an opportunity to evaluate whether anthropometry plays a role among the first draft of selected players. In both cohorts, goaltenders were removed from the study, due to their specific tasks. 

#### 2.1.2. Variables and Instruments—Camp 1

Anthropometric measures are used with the objective of body composition assessment of ice hockey players [28,29,30,31,32,33]. Many researchers have put attention on lower limb power, which can be related to skating performance in different areas (e.g., on-ice acceleration, on-ice speed, etc.). Indeed, tests such as the horizontal and vertical jumps are often cited and they are parts of the NHL Combine and in international team evaluation protocols [28,31,35,45]. Based on that, we selected the standing broad jump and the counter movement jump as our performance tasks for evaluation. Ice hockey also stresses the shoulder girdle, where shoulders receive hits or contacts, and the arms are responsible for controlling, passing, shooting the puck, and battling for the possession. The physical qualities of the upper body such as strength, power, and endurance have been studied in relation to performance [51]. Based on that, we selected three tests to assess these attributes: (1) grip strength, which is considered as a classic fitness test in ice hockey [44], (2) the seated medicine ball throw [52], and (3) the chin-up (max repetitions) [38]. Considering the intermittent nature of ice hockey and its high physiological demands, we identified three variables that are frequently associated with hockey [30,49]: (1) aerobic capacity, (2) agility, and (3) sprinting. These variables are often found in scientific literature, as well in the culture of professional clubs and international teams. Being consistent with this evaluation culture, aerobic capacity was evaluated with the Léger 20 m shuttle run test [53], a valid and reliable test for adolescent hockey players. The pro agility test, also called the 5-10-5, also part of the NHL Combine, was used to assess the change of direction capacities of players [54]. Finally, a 30 m running sprint completed the running evaluation, giving our evaluation the possibility to compare with the sprint test on ice. Many researchers have analyzed specific on-ice variables: skating acceleration, top speed, and skating agility are all variables that have the potential to predict performance [12,17,30,35]. For our purposes, the 44.8 m skating sprint was chosen in the testing plan [17] as well as the skating agility test of the Finnish International Ice Hockey Centre of Excellence of Vierumaki [55], a skating sequence which requires the player to effectively perform a wide range of movements in the ice hockey player’s repertoire (e.g., sharp turns, pivots, and shorts sprints in both forward and backward skating).

### 2.2. Measures—Camp 1

#### 2.2.1. Anthropometric Measures and Off-Ice Fitness Protocols

Measures were selected in collaboration with the federations’ stakeholders and align with specific components identified as potential determinants of performance in ice hockey. For anthropometry, height and mass were assessed. For off-ice fitness, nine tests were selected and divided into three categories: (1) lower limb power, (2) upper limb strength-power-endurance, (3) running speed, agility, and VO2max. On-ice tests were used to measure skating speed and agility. Table A1 in Appendix A describes more specifically the protocol that was implemented. After completing the anthropometric measures, a warm-up was conducted by a certified strength and conditioning coach before the evaluation session. The 15-min warm-up combined a running activation, ballistic movements, light footwork, twenty-meter shuttle runs, low-intensity plyometrics, core activation, and upper limb mobility exercises. Both females and males were divided into four groups. Each group was divided in pairs or trios, rotating to each of the evaluation stations to complete the full battery of neuromuscular tests. In order to maintain consistency in the evaluation process, each evaluation station had the same evaluator through the entire process. Aerobic capacity was assessed at the end of each group’s evaluation, to so that the energy cost of the test did not interfere with and influence the other results.

#### 2.2.2. On-Ice Skating Abilities

**Figure 2:**
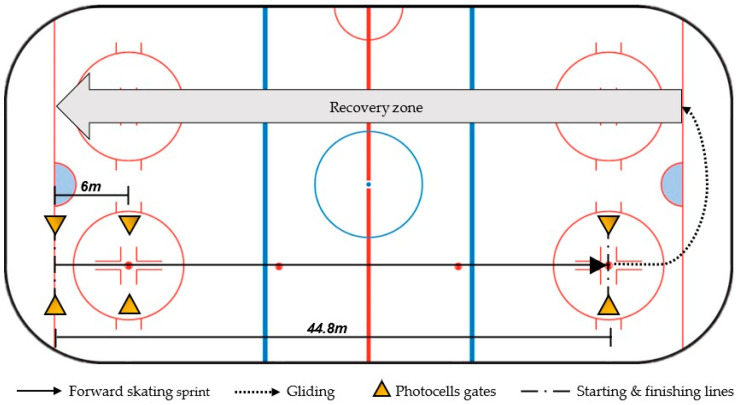
The 44.8-m skating sprint test.

**Figure 3:**
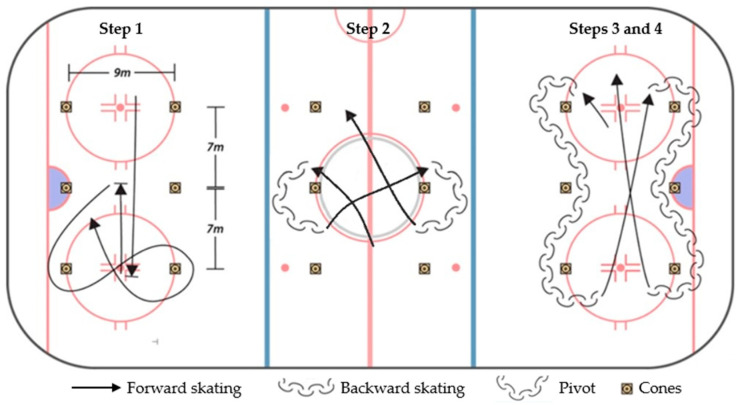
The skating agility test, with the four-step procedure.

Figure 2 and Figure 3 illustrate on-ice tests that were conducted. Prior to the on-ice evaluation session, a warm-up was administered and supervised by the Camp 1 coaches. The warm-up consisted of a 10-min session combining a light forward-skating warm-up, on-ice mobility, skating drills, individual cardiovascular solicitation, and short on-ice acceleration/deceleration drills.

Figure 2 illustrates the set-up of the forward-skating sprint test. The athlete is in a standing position with one foot behind the starting line. The athletes begin their sprint as soon as they wish. The athlete skates as fast as he/she can from the start to the end of 44.8 m distance in a straight line. The starting stance is left free to the athletes to decide; they are instructed to decelerate only after the sprint distance has been covered. Two attempts are made to achieve the best time, each separated by a 3-min passive recovery on the ice.

As shown on Figure 3, the skating agility circuit was conducted in four phases (on the same circuit). Phase 1: The athletes stand with their feet placed behind the starting line. When the photocell gate turns green and beeps, the athletes can start the test as soon as they wish. The athletes first skate from one end of the circuit to the other in a straight line, brake, then accelerate back to the center line to the second pair of cones and brake again. They then skate to their left, outside the cone, to make two consecutive short turns around the two cones of the third pair. Phase 2: After the short turns, the athletes skate to the second pair of cones, where they perform two open pivots (facing the direction of the starting line) around the cones. Phase 3–4: After the open pivots, the athlete skates to the first pair of cones. As the athletes rotate around the first cone, they pivot completely and skate backwards to the third pair, performing a slalom with the second pair. After completing one side of the backward skate, they advance to the other side diagonally and repeat the pivot for the backward slalom on the opposite side. After completing the second side, the athletes sprint to the start line. The best of the two trials is recorded, separated by a five-minute recovery.

### 2.3. Measures—Camp 2

#### 2.3.1. Male Cohort: Anthropometry and Match Performance

Male players were only involved in a tournament-showcase in which they played three matches (3 × 3 (20 min) periods) at Camp 2. No on-ice testing was conducted with this group. Upon arrival at the competition, we assessed athletes’ body mass and height. All matches were video-recorded, and match performance data were collected by using the InStat^®^ platform [56]. We chose six indicators (divided into three components) that reflect multiple facets of match performance in ice hockey: (1) physical implication, (2) offensive contribution, and (3) defensive contribution (see Table 1). For physical implication, we determined that the number of body checks given and received (e.g., hits, hits against) would reflect a player’s physical implication. We measured offensive contribution by players’ Expected goals (xGs) per game and Corsi %. Expected goals (xG) is an InStat calculated statistic that is calculated using an algorithm that takes into account shot location and other parameters such as match situation and opponent’s goaltender efficiency. Corsi % is a “puck possesion” metric, which refers to a player’s performance in terms of puck possession. Corsi is the sum of a player’s shots towards the opponent’s net (e.g., shot on net, missed shots, blocked shots). Corsi against is the same metric but calculated from the opponent’s performance. Corsi % is derived from the proportion of a player’s Corsi (for) related to the total amount of Corsi scores (for + against). We assessed defensive contribution by using Opponent expected goals (D-xG) and shot blocking, which refers to a player’s involvement in the defensive zone.

#### 2.3.2. Female On-Ice Skating Abilities and Match Performance

For the female evaluation camp, we assessed body height–mass upon arrival at the camp. On day 1, we tested on-ice skating abilities during the first on-ice session. We applied Camp 1 protocol in which we measured skating speed-acceleration and agility; in addition to that we added the 44.8-m backward skating test, following the same procedure as the forward skating sprint test. Match performance was assessed during intra-squad matches. Due to the context (setting and structural organization) of Camp 2, Instat^®^ platform was only in charge of male analytics official matches. As a consequence, we collected match performance manually, by using an observational grid, for female intra-squad matches. We chose four performance markers, which were most similar to those analyzed by the Instat^®^ platform, that can be performed by both forwards and defense players: (1) physical implication, (2) puck recoveries, (3) offensive actions, and (4) shot attempts. Physical implication was defined as every action in which players were involved in puck battles (e.g., along boards, hits for and hits against). Puck recoveries refers to every situation in which a player recovers a loose puck and maintains possession. Offensive actions were defined as any action that contributed to puck possession such as passes to teammates and zone entries. Shot attempts were defined as all of a player’s shots towards the opponent’s net. Finally, blocked shots were removed from the database because these actions were only collected for players who played on defense (too many 0 values for forwards). To assure reliability, two evaluators were involved in the coding process. Video-recorded matches were observed two times, until inter-evaluator agreement was congruent (≥90% of agreement).

### 2.4. Statistical Analyses 

All statistical analyses were conducted with SPSS software (version 28; Chicago Illinois, USA). We verified for normality assumptions for each variable under study, using Kolmogorov–Smirnov (K-S) test for normality and verified skewness and kurtoses values for each distribution. No violation of normality was observed (all K-S between 0.086 and 0.234; all at *p* > 0.50), except for two match performance indicators (e.g., males’ xG, shot blocking; females’ physical implication). However, the skewness values of these markers were still under 1.0 which is deemed acceptable for correlational analyses [57]. Descriptive statistics were calculated for each variable to provide an overview of the sample under study. For the first objective of the study (evolution of anthropometric measures between two camps), we performed paired t-tests by comparing height and mass scores among those who took part in both evaluation camps. For the second objective (associations between anthropometry and performance), we calculated Pearson correlation coefficients involving Camp 1 anthropometric measures (e.g., height and mass), off-ice fitness, and on-ice skating tests. We decided to verify for associations that were related with anthropometry, in this regard, associations between fitness and match performance were beyond the scope of this study. For Camp 2 measures, we computed Pearson correlation coefficients and verified the associations between anthropometric measures and match performance for males. In the female cohort, we verified the associations by analyzing correlation coefficients between anthropometric measures and results from the on-ice skating tests that were performed at camp. Due to the smaller sample that resulted from the first selection process (Camp 1), we decided to focus more on associations that displayed significant associations. Therefore, we classified height and body mass in ascending order and calculated mean scores for each group of five athletes. We produced a graphic representation between these sub-scores and their corresponding values in match performance (males) and skating agility tests (female). For the third objective of the study (player’s position), we calculated partial coefficient correlations by controlling player position (forward vs. defense) in each series of analyses.

## 3. Results

### 3.1. Descriptive Statistics

Table 2 displays descriptive statistics at both evaluation camps. No significant differences were observed when comparing selected male players versus those who were cut at Camp 1. We observed significant increases in the selected males’ height and mass between the two evaluation camps: t_height_ = 4.20; *p* < 0.001: h^2^ = 0.70; t_mass_ = 2.55; *p* < 0.05: h^2^ = 0.43. In the female cohort, there were higher scores in fitness and skating ability, when compared to players who were cut at Camp 1. A significant increase in body height was observed among females, but not in body mass: t_height_ = 2.79; *p* < 0.01: h^2^ = 0.42; t_mass_ = 1.93; *p* = 0.06: h^2^ = 0.29. In both cohorts, no significant differences were observed regarding players’ position and their anthropometric profile. The correlation between body mass and weight was significant at Camp 1: r_male_ = 0.790; r_female_ = 0.457; *p* < 0.001. At Camp 2, similar correlations were observed for both groups: r_male_ = 0.775; r_female_ = 0.518; *p* < 0.001.

### 3.2. Camp 1

#### 3.2.1. Anthropometry and Off-Ice Fitness

Table 3 shows Pearson correlation coefficients for male players in Camp 1. Results revealed that taller male players displayed higher performance in the broad jump (r = 0.268; *p* < 0.05), produced more absolute force and power during vertical jump (r = 0.540; *p* < 0.01 and r = 0.637; *p* < 0.001), and had higher eccentric and concentric impulse capacities during jumping (r = 0.493; *p* < 0.01 and r = 0.653; *p* < 0.01). They also had better absolute grip strength (r = 0.501; *p* < 0.001). Taller players were also faster in the 30 m sprint test (r = −0.313; *p* < 0.001). Similar patterns were also observed regarding the associations between body mass and off-ice fitness. Heavier players demonstrated higher performance in broad jump (r = 0.230; *p* < 0.05), produced more absolute force and power during vertical jump (r = 0.679; *p* < 0.001 and r = 0.813; *p* < 0.001), and had higher eccentric and concentric impulse capacities during vertical jumping (r = 0.617; *p* < 0.001 and r = 0.856; *p* < 0.001). They also developed more absolute grip strength (r = 0.595; *p* < 0.001) and were also faster (r = −0.246; *p* < 0.05).

For female players, results revealed that taller players displayed higher performance in absolute grip strength (r = 0.219; *p* < 0.05) and had higher eccentric and concentric impulse capacities during vertical jump (r = 0.189; *p* < 0.05 and r = 0.275; *p* < 0.01). However, taller female players demonstrated poorer performance when we focused on other parameters, such as relative maximal power (−0.268; *p* < 0.01) and vertical jump flight time (r = −0.214; p < 0.05). Heavier female players demonstrated lower performance in the broad jump and vertical jump (respectively, r = −0.290 and r = −0.267; *p* < 0.001), as well as relative maximal power (−0.389; *p* < 0.001) and vertical jump flight time (r = −0.316; *p* < 0.01). Regarding speed capacity, results showed that heavier female players were slower in sprint time (r = 0.314; *p* < 0.001), slower in change of direction time during 5-10-5 pro-agility test (r = 0.263; *p* < 0.05), and also demonstrated that they had less VO2max (r = −0.322; *p* < 0.001), However, when we focused on upper body absolute force output, heavier players had higher performance results (r = 0.376; *p* < 0.001), and also displayed better absolute grip strength (r = 0.227; *p* < 0.05).

#### 3.2.2. Anthropometry and On-Ice Fitness at Camp 1

Table 4 shows Pearson correlation coefficients for male and female players in Camp 1. Results revealed that taller male players displayed poorer on-ice agility performance at the skating agility test (r = 0.266; *p* < 0.05). A similar pattern was observed for heavier players, regarding the association between body mass and on-ice agility test (r = 0.316; *p* < 0.01). Results from the female cohort revealed no correlation between height and on-ice performance. However, heavier female players displayed poorer on-ice acceleration and forward skating sprints (respectively, r = 0.206; *p* < 0.10 and r = 0.257; *p* < 0.05).

### 3.3. Camp 2

#### Male Cohort In-Depth Analysis: Anthropometry and Match Performance

Table 5 shows Pearson correlation coefficients for male players in Camp 2. Results demonstrated only significant associations between body mass anthropometry characteristics and shot blocking, a negative correlation meaning heavier male players tend to display less shot blocking (r = −0.399; *p* < 0.05).

**Figure 4:**
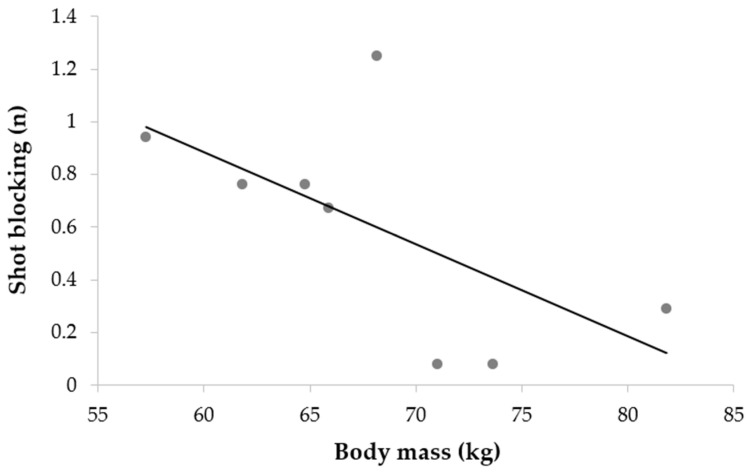
Association between body mass and shot blocking (game blocked shots).

**Tendencies on shot blocking and anthropometric body mass**. Since the only significant association was between shot blocking and body mass, we took a more specific approach to this association. Figure 4 shows that the association between the classified body mass score and shot blocking was significant (r = −0.647; *p* < 0.01). These results suggest that heavier players displayed less efficiency in that category of defensive action.

### 3.4. Camp 2: Female Cohort

#### Female Cohort In-Depth Analysis: Anthropometry and Performance (Skating and Match Performance)

Table 6 shows Pearson correlation coefficients for female players in Camp 2. Results demonstrated no significant associations between anthropometry characteristics (height or mass) and on-ice skating performance, except for a favorable trend between height and skating agility (*p* < 0.10).

**Figure 5:**
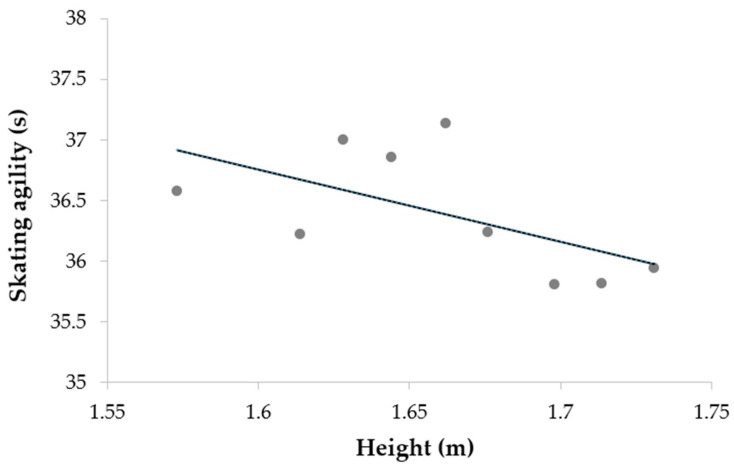
Association between height and skating agility test (seconds).

**Tendencies of associations among the height-body mass classified sub-groups**. Since we found only one significant association (at *p* < 0.10) between anthropometric measures and skating performance, we verified the tendencies for associations between height and on-ice skating performance at Camp 2 by considering the lesser level of variability among the selected players from Camp 1. Figure 5 shows that taller players have better scores in skating agility (r = −0.585; *p* < 0.05).

## 4. Discussion

In ice hockey, talent identification and selection camp processes are complex. Professionals evaluate physical fitness, on-ice skills, and match performance. According to Ransdell et al. [38], anthropometric characteristics have an impact on the performance of ice hockey players, with athletes from the most successful international teams being taller, heavier, and more muscular. This study sheds light on the impact of anthropometric characteristics on the selection camp phases of off-ice fitness, on-ice testing, and match performance in young Canadian elite ice hockey athletes.

The first part of our study examined the follow-up selection camp processes and anthropometric evolution over a five-month period. Our results suggest a height and body mass increase in male hockey athletes during this short period of time. Body mass results were corroborated by Leiter et al. and Cordingley et al. [9,39], who have observed similar body mass positive trends over this peak growth period (e.g., 13–15 years old). Our results about body height trends are similar to Cordingley et al. [39], especially with male athletes, but different from those of Leiter et al. [9] which showed no evolution over a six-month to one-year period between fourteen to sixteen years old. This difference in results could be explained by the fact that our population was young Canadian top elite athletes, a relatively homogeneous group, while those from Leiter et al. came from diverse ice hockey teams across the country, with an heterogenous mix of levels. An anthropometric evolution over a five-month period could be explained by a growth spurt and maturation because young male athletes were fourteen years of age [11]. The relative age effect bias reason [12], meaning that athletes selected from Camp 1 to Camp 2 were taller and heavier to begin with even during Camp 1, was excluded because no significant differences were observed when comparing selected male players versus those who were cut at Camp 1. In contrast, the anthropometric evolution of young female hockey players only demonstrated a minimal height increase, while body mass remained stable over the five-month period. To the best of our knowledge, no studies have observed the anthropometric evolution of young female top elite ice hockey players. A small height increase and body mass linearity could be explained by some factors: (1) inverted relative age effect bias, female athletes selected from Camp 1 to Camp 2 were already leaner in Camp 1, resulting in lowering the average [12]; (2) growth spurt period had already passed, because athletes in this group were sixteen years old and so many changes had already occurred before this age [39].

The second part of the present study was related to relationships between anthropometric measurements and performances. Our results suggest the relative importance of anthropometric variables in the early phases of detection and selection at Camps (e.g., Camp 1 in our protocol) on off-ice fitness parameters and on-ice performance, more than in the latter phases (e.g., Camp 2). In the first phases of selection, anthropometry could have played an important role on off-ice performance. Taller and heavier male youth ice hockey players demonstrated better performance in many components (horizontal and vertical jump, grip strength, acceleration, and speed), where some results were not significant (change of direction, aerobic capacity, upper body power). Our significant results corroborated with previous studies [40,49,58] whereas anthropometry was related to some but not all off-ice fitness in youth and collegiate male hockey players. One result came more specifically to our attention: the significance of anthropometry on absolute maximal power produced during the vertical jump, while being insignificant on relative maximal power. It could be interpreted that body mass plays an important role on absolute off-ice variables, whereas heavier players perform better because of their larger body mass. However, when we look at these relations with relative variables, the correlation faded away. In this population, it would therefore be relevant to evaluate both absolute and relative data to take an objective look at off-ice fitness performance. For females, however, interpretation of the results was different: a positive correlation between anthropometry and absolute maximal power was produced during vertical jump, while this correlation became negative with the same variable transformed in relative values. A possible explanation is that body mass (and height to a lesser extent) could be detrimental to performance. We could interpretate theses result as young female athletes who had higher body mass likely having more body fat, explaining better performances with absolute values and poorer performance after transforming them into relative values. Nevertheless, body mass has a negative impact on off-ice performance among young female hockey athletes. We found that heavier female athletes tended to perform more poorly than their leaner teammates on some tests such as aerobic capacity, sprint, and jump, confirming what Gilenstam and colleagues found before us with older Swedish female hockey athletes [50]. Positive correlations existed between on-ice performance and anthropometry characteristics (e.g., mainly body mass) in our study, leading us to interpret anthropometry as a “negative” factor for on-ice performance. As it was for on-ice agility in male athletes, and on-ice acceleration and speed capacities for females, our results concurred with previous studies which looked at anthropometry and on-ice performance relationships [29,30,40,58]. They all found small correlations between body mass and on-ice performance, finding that being heavier was detrimental to acceleration and skating speed. However, our correlations seem to disappear from Camp 1 to Camp 2 on-ice testing results comparison with anthropometry in female athletes. It could be explained by the more homogeneous selected athlete group; athletes with a less advantageous morphology for performance on ice were cut by the Hockey Québec coaching staff after Camp 1. Some correlations with off-ice or on-ice results were observed in Camp 1, which seemed to fade away in Camp 2. As the athletes’ group became smaller between the two camps, performance capacity differences followed the same patterns by narrowing. Consequently, fewer differences could be observed as the overall level became more homogenous. The role of anthropometry (especially body mass) for on-ice performance has already been examined by Lemoyne et al. [14], with their anthropometric model which was non-significant unlike the skating performance model. Unlike previous studies and our previous results, when data were analyzed with a sub-group classification, we observed that being taller and heavier were advantageous for on-ice performance for the female group in Camp 2.

Our study illustrated the limitations of the relationship between anthropometry and match performance. Indeed, we showed that only the shot blocking parameter was related to body mass in young male athletes, heavier athletes were less capable of blocking shots from their opponent. With the sub-group analysis, we observed the tendency of lighter athletes to block more shots. The lack of correlation between anthropometry and ice hockey match performance has already been highlighted by several studies [7,29,58]. Boland et al. [29] found no correlations between anthropometry and game performance, even if evaluators looked at “simple” indicators (e.g., goals, assists, plus/minus). Congruent with our results, Stanula and colleagues found that anthropometric variables were not related to match performance indicators among elite Polish hockey players [58]. Delisle-Houde and colleagues found correlations between body composition characteristics (e.g., body mass and body fat percentage) and ice-time metrics in elite male collegiate Canadian hockey players [7]. They showed that heavier players tend to spend more time on the ice during shifts and shot more on the net. In this regard, a more holistic approach seems to have emerged, suggesting the importance of evaluating a combination of physical, technical, psychological, and match performance components in the talent identification process [14].

### Limitations and Future Perspectives

Despite its contribution, the present investigation also has its limitations. The first concerns the assessment protocol for anthropometric measures. In order to be time-efficient during these camps, we chose to evaluate only body mass and height, as other measures were prioritized and took more time to assess (i.e., off-ice fitness and on-ice testing). Despite the financial and time costs of DEXA or skinfolds methods, these are both more accurate than traditional evaluation. Indeed, our simple method is time-group-camp-effective, but less precise, especially for body mass assessment. We believed that with a more precise evaluation tool, results would have provided a more precise picture of the associations between body composition and performance [15]. Body fat percentage was the anthropometric parameter most related to on-ice performance, according to Delisle-Houde et al. [7]. More precise anthropometric parameters will be assessed in subsequent phases of the Hockey Quebec ice hockey selection process (e.g., Camps 3 and 4) once the team has been finalized. Further analysis will consider anthropometric variables more accurately, such as body fat percentage, to explain off-ice, on-ice, and match performance differences. Similar anthropometric protocols were used to evaluate adolescent players at other international selection camps because of their possible growth spurt during the maturation phase that caused high body mass and height gains in a few short months [11]. It is logical, in an adolescent evaluation process, to focus more on physical and technical assessment tools than anthropometrics, because the latter could vary greatly within short periods of time, as a result of maturation and growth spurts. A second limitation was the potential selection bias, because to participate in the study, all players had to be registered in a league governed by Hockey Quebec. This means that participants were engaged in a very similar pattern of sports development, possibly leading to an almost identical athletic profile, reducing the discriminant capacity of the protocols. Another limitation concerns the physical maturation stage issues of players (early birth vs. late birth) on performance outcomes [12]. The maturation process is an important factor to consider, especially in late maturing team sports like ice hockey, where physical and anthropometric attributes impact technical performance. We did not analyze the impact of birth date quartiles on performance, but we know that there are recruitment biases in ice hockey, as the literature has already dealt with this topic [59]. Further analysis in relation to relative age or maturation stage could have helped us better understand and interpret differences. Another limitation was concerning the protocol differences for collecting match performance data in both cohorts. In Camp 2, female cohort was held with intra-squad matches, without access to In-stat^®^ analytics data, unlike the male cohort tournament. In this regard, we decided to collect data manually for female athletes, trying to get as close as possible of Instat^®^ match performance indicators used for male cohort. We knew it would lead to a limitation when comparing associations between anthropometry and match performance between gender. Further analysis could consider match performance indicators with InStat^®^ platform for both female and cohort, to better understand gender differences. We also decided to remove blocked shots indicator from female match performance analysis, which another limitation, while keeping this indicator for male cohort. Results suggested that this parameter was related to body mass in male athletes, so we could imagine it could also be linked to anthropometric characteristics in female athletes. More accurate objective on-ice and match measures, such as global or local positioning system technologies (e.g., GPS or LPS), could contribute to better understand the role of anthropometry on precise match performance indicators (e.g., peak of accelerations/decelerations, skating speeds, explosive efforts, or strides).

## 5. Conclusions

This article highlights the importance of evaluating anthropometrics in selection camps for young elite ice hockey athletes. It helps us to better understand the relationship between anthropometry and functional performance in off-ice, on-ice, and match situations during talent identification camps. Correlations exist, but after the second phase of the selection process, the effect was reduced. As young ice hockey athletes complete the selection process, the relative importance of an “optimal” anthropometric profile beyond performance in off-ice fitness, on the ice, and match performance, seems to decrease. The assessment of anthropometric characteristics should not take precedence during these adolescent growth phases, as other qualities are more important to evaluate, such as game intelligence or technical capacities on the ice. Following the divergent results obtained, we invite organizations and federations to be cautious when analyzing anthropometrics, and their relation to performance in adolescents or the importance given to the physical qualities of a player.

## Figures and Tables

**Table 1:** Match performance indicators, collected with the InStat^®^ platform.

Performance Marker	Definition
Hits against	Situations in which a player is hit by an opponent.
Hits	Situations in which a player hits an opponent (shoulder check).
Expected goals with player on (xG-on)	Probability of scoring goals when the player is on the ice. Reflects the potential offensive contribution of a player.
Corsi %	Amount of shot attempts related to opponents’ shot attempts (player’s shot attempts + opponent’s shot attempts). Higher % means that player contributes to their team’s puck possession.
Opponent expected goals (D-xG)	Probability of seeing an opponent score when the player is playing in the defensive zone. Higher scores mean that a player is not contributing to their team’s success.
Shot blocking	Sum of situations in which a player blocks the opponent’s shot to the net.

**Table 2:** Descriptive statistics (Camp 1 and Camp 2).

Variables	Male Cohort		Female Cohort	
	Camp 1 (*n* = 80)	Camp 2 (*n* = 41)	Camp 1 (*n* = 107)	Camp 2 (*n* = 48)
Age	13.81 ± 0.40	14.21 ± 0.41	14.96 ± 0.92	15.69 ± 0.87
Anthropometry				
Body Height (m)	1.72 ± 0.076	1.76 ± 0.068 **	1.65 ± 0.058	1.66 ± 0.049 **
Body Mass (kg)	64.62 ± 9.30	68.24 ± 8.82 **	62.75 ± 8.79	63.77 ± 7.38
Off-ice fitness				
Broad jump (m)	2.26 ± 0.014	n/a	1.95 ± 0.015	n/a
Vertical jump (m)	0.48 ± 0.066	n/a	0.44 ± 0.061	n/a
Flight time (s)	0.31 ± 0.04	n/a	0.24 ± 0.04	n/a
Absolute maximal power (W)	3059.19 ± 546.73	n/a	2483.95 ± 353.05	n/a
Relative maximal power (W/kg)	47.13 ± 4.75	n/a	39.80 ± 4.58	n/a
Concentric mean power (W)	1587.51 ± 323.34	n/a	1287.16 ± 193.70	n/a
Eccentric impulse (ms)	75.48 ± 16.82	n/a	68.50 ± 12.73	n/a
Concentric impulse (ms)	164.51 ± 26.57	n/a	141.75 ± 20.66	n/a
Grip strength (kg)	95.83 ± 16.79	n/a	72.56 ± 13.40	n/a
Chin-ups (*n*)	8.93 ± 3.71	n/a	3.07 ± 2.95	n/a
Medicine ball throw (W)	169.64 ± 26.08	n/a	134.80 ± 18.39	n/a
MB throw relative power (W/kg)	8.01 ± 1.86	n/a	6.10 ± 1.11	n/a
30 m sprint (s)	4.78 ± 0.20	n/a	5.05 ± 0.23	n/a
5-10-5 Pro agility (s)	5.31 ± 0.19	n/a	5.65 ± 0.26	n/a
VO_2_max (mL/min/kg)	49.72 ± 4.86	n/a	44.28 ± 5.49	n/a
On-ice skating				
0–6 m acceleration (s)	1.31 ± 0.07	n/a	1.44 ± 0.08	1.36 ± 0.06
44.8 m forward skating sprint (s)	6.27 ± 0.23	n/a	6.85 ± 0.23	6.44 ± 0.20
Skating agility (s)	35.42 ± 1.29	n/a	38.63 ± 1.91	36.40 ± 0.94
0–6 m backward acceleration (s)	n/a	n/a	n/a	1.62 ± 0.09
44.8 m backward skating sprint (s)	n/a	n/a	n/a	7.81 ± 0.36
Match performance indicators (per game)				
Hits (nb)	n/a	0.24 ± 0.31	n/a	n/a
Hits against (nb)	n/a	0.47 ± 046	n/a	n/a
xG with player	n/a	0.49 ± 0.44	n/a	n/a
Corsi for %	n/a	0.51 ± 0.08	n/a	n/a
Opponent xG	n/a	0.28 ± 0.24	n/a	n/a
Shot blocking	n/a	0.56 ± 0.67	n/a	n/a
Physical implication	n/a	n/a	n/a	0.76 ± 0.88
Puck recoveries	n/a	n/a	n/a	1.35 ± 1.17
Offensive actions	n/a	n/a	n/a	5.89 ± 2.99
Shot attempts	n/a	n/a	n/a	4.62 ± 2.48

** Significant increases between Camp 1 and Camp 2 anthropometric measures (*p* < 0.01).

**Table 3:** Associations between anthropometry and off-ice fitness at Camp 1.

	Males (*n* = 80)		Females (*n* = 107)	
Test	Height	Body Mass	Height	Body Mass
Broad jump	0.268 *	0.230 *	0.069	−0.290 ***
Vertical jump	0.099	0.009	−0.147	−0.267 ***
Flight time	−0.002	0.026	−0.214 *	−0.316 ***
Absolute maximal force	0.540 **	0.679 ***	0.131	0.481 ***
Absolute maximal power	0.637 ***	0.813 ***	0.151	0.569 ***
Relative maximal power	0.096	0.0106	−0.268 **	−0.389 ***
Concentric mean power	0.487 ***	0.690 ***	0.151	0.562 ***
Eccentric impulse	0.493 **	0.617 ***	0.189 *	0.468 ***
Concentric impulse	0.653 **	0.856 ***	0.275 **	0.693 ***
Grip strength	0.501 **	0.595 ***	0.219 *	0.227 *
Medicine ball throw	0.048	0.179	−0.043	0.376 ***
MB throw relative power	−0.071	−0.079	−0.060	−0.089
30-m sprint	−0.313 **	−0.246 *	0.015	0.314 ***
5-10-5 Pro agility	−0.095	0.068	0.067	0.263 *
VO_2_max	−0.168	−0.053	−0.048	−0.322 ***

* *p* < 0.05; ** *p* < 0.01; *** *p* < 0.001.

**Table 4:** Associations between anthropometry and on-ice fitness at Camp 1.

	Males (*n* = 80)		Females (*n* = 107)	
Test	Height	Body Mass	Height	Body Mass
0–6 m acceleration	−0.095	−0.094	0.031	0.206 ^†^
44.8 m forward skating sprint	−0.179	−0.137	0.210	0.257 *
Skating agility	0.266 *	0.316 **	0.075	0.137

^†^*p* < 0.10; * *p* < 0.05; ** *p*< 0.01.

**Table 5:** Associations between anthropometry and match performance indicators (InStats)**.**

Test	Height	Body Mass
Hits	0.125	0.231
Hits against	0.036	0.010
xG	0.142	−0.084
Corsi %	−0.081	−0.087
Defensive xG	0.088	−0.144
Shot blocking	−0.255	−0.399 *

* *p* < 0.05.

**Table 6:** Associations between anthropometry and on-ice fitness.

Test	Height	Body Mass
0–6 m acceleration	−0.018	0.120
44.8 m forward sprint	0.031	0.065
0–6 m backward acceleration	0.180	0.161
44.8 m backward sprint	−0.008	0.051
Skating agility	−0.248 ^†^	−0.107
Physical implication	0.061	0.014
Puck recoveries	0.171	0.087
Offensive actions	0.213	0.151
Shot attempts	0.115	0.088

^†^*p* < 0.10.

## Data Availability

Data in the actual form were used by researchers and collaborators in the actual project. Researchers agreed to give access to data upon request. Those interested should inform the corresponding author at: gaetan.martini@uqtr.ca.

## Appendix A

**Table A1:** Anthropometric and off-ice fitness tests explained.

Test	Measures (Units) (Instrumentation)	Protocol
Anthropometry Height	Body height (m)	Stand on the stadiometer platform with shoes off, with feet together, arms alongside the body. Then gaze outward; the measurement was taken following a maximum respiration rate to the nearest 0.5 cm.
Anthropometry Body Mass	Body mass (kg)	Instruction to step on the scale, with shorts, tee-shirt, and shoes off. Results are collected in kilograms, to the nearest 0.1 kg.
Standing broad jump	Length of jump (m) (Tape on floor)	Stand behind a marked line on the floor, with feet hip-width apart. Instruction to jump as far as possible, by quickly bending/extending legs and swinging arms back and forth, landing on both feet without falling. Foot that travelled the shortest distance is measured. The best of three attempts is selected.
Vertical jump	Height of jump (m) [Vertec] (Power Systems, Knoxville, TN, USA)	Stand under the testing device to measure maximum height with arms fully extended. Then, position self at an upper arm’s distance from the testing device and perform one pre-trial jump. Bend legs and pause for a second before jumping while reaching out as high as possible, by using arms. The best of three attempts is retained.
Counter movement jump	Flight time (s) Peak power (W) Relative peak power (W/kg) Concentric mean power (W) Eccentric impulse (ms) Concentric impulse (ms) (Dual Pasco 2-Axis Force Platform S-2142, 1000 Hz) (Roseville, CA, USA)	Stand immobile, feet on the device. Once body mass is recorded, athlete is advised to follow supervisor instructions before starting jumping. Athlete is instructed to start in standing position and must keep hands on hips during the whole assessment. They lower their center of mass by quickly bending their legs and hips, then braking suddenly (approximately at 90° knee flexion), produce force as quickly as possible to extend legs and jump vertically. No snap down (feet takeoff during lowering the position) is allowed. The best of two attempts is retained.
Grip strength	Sum of grip test (kg) (Jamar^®^ hydraulic hand dynamometer) (Performance Health, Chicago, IL, USA)	Dynamometer is set to zero before each test and adjusted to athlete hand. Grab dynamometer in a neutral grip and keep it close to hips. Exhale heavily and compress it as hard as possible during three to five seconds. Then give back the device to the evaluator who records the result and resets it at 0 for the next test. Two attempts per hand are allowed, best result per side is retained.
Seated medicine ball-throw	Power (W) Relative power (W/kg) (Move Factor Ballistic Ball™) (Assess2Perform, Petoskey, MI, USA)	Sit on the floor with the back against the wall and feet hip-width apart. Ball is placed against the chest, and held at the sides and behind the center, with the forearms parallel to the floor. Throw the ball as hard as possible while keeping the back against the wall. The ball’s accelerometer records the throwing parameters. The best of the three attempts is selected.
Chin-ups	Maximum reps (n) (Bar for hanging)	Hang from the bar with hands shoulder-width apart with a supinated grip, arms fully extended. Pull-up by bending arms until the chin passes over the bar, then lower to the starting position. Leg swings are not allowed. Only reps performed with the correct technique are counted.
30-m sprint	Time (s) (Swift Speedlight photocells) (Swift Performance, Northbrook, IL, USA)	Stand with feet behind the starting line. Initiate the sprint at any time after evaluator signal. Sprint as quickly as possible to the finish line. Should decelerate only after crossing the sprint distance to obtain the best possible time. Two attempts separated by a 3 min break; best time is retained.
5-10-5 Pro agility	Time (s) (Swift Speedlight photocells) (Swift Performance, Northbrook, IL, USA)	Place feet on middle starting line. Begin by moving as quickly as possible to a line from the end past the photocells and touch the line with one hand, then move as quickly as possible to the line at the other end, touch the line with one hand before returning to the center. Best of two attempts is retained.
Léger 20 m Shuttle run	Maximum oxygen consumption (mL·kg^−1^·min^−1^) (Tool kit)	Repetition of 20 m shuttle runs to maximal fatigue. Must stand 1 m behind the line before the next beep before changing direction and continue to run, then must wait for the signal before starting. The speed increases with each level. Follow the rhythm of the soundtrack. One trial is recorded.
